# Classification and Treatment Strategies of Concomitant Fibular Column Injuries in Tibial Plateau Fractures

**DOI:** 10.1155/2021/2698642

**Published:** 2021-09-06

**Authors:** Xiang Yao, Bin Lv, MinJie Hu, Jishan Yuan, Xiaochen Fan, Kaihua Zhou, JiLei Tang, Lei Wang

**Affiliations:** ^1^Department of Orthopaedics, The Affiliated People's Hospital of Jiangsu University, Zhenjiang, Jiangsu Province 212000, China; ^2^Department of Orthopaedics, Qingpu Branch of Zhongshan Hospital, Fudan University, Shanghai 201700, China; ^3^Department of Orthopaedics, Affiliated Qidong Hospital of Nantong University, Nantong 226200, China

## Abstract

**Background:**

About 1/3 of tibial plateau fractures are associated with proximal fibula fractures, but most proximal fibula fractures are often ignored. The aim of this study was to precisely explain the classification and treatment strategies of six injury types of the fibular column associated with tibial plateau fractures.

**Methods:**

Patients with ipsilateral proximal fibula and tibial plateau fractures treated in our hospital were retrospectively reviewed from Aug 2007 to Mar 2020. Two experienced surgeons and two radiologists divided fibular column injury into 6 injury types according to the AO classification and four-column nine-segment classification. The treatment scheme (surgically treated or conservatively treated) was also recorded.

**Results:**

In total, 355 proximal fibula fractures were included. Type 2 fibular head fracture was the most common type of injury in 122, and the segregate of superior tibiofibular syndesmosis was the rarest type in 3. In avulsion injury proximal of fibular pattern, the proportion of patients who need surgical intervention is the highest.

**Conclusions:**

Six injury types in the four-column nine-segment classification covered all types of bony and soft tissue injuries of the fibular column and concisely explained the injury mechanism. The classification is helpful for the precise judgement and decision-making of the concomitant fibular column injuries in tibial plateau fractures.

## 1. Introduction

The proximal fibula is close to the posterolateral tibial plateau and is closely connected by the superior tibiofibular syndesmosis. The proximal fibula provides functional stability to the posterolateral structures of the knee and plays a crucial role in the anatomy and clinical function of the knee. The PLC is composed of the fibular collateral ligament (FCL), biceps femoris tendon (BFT), popliteus tendon, and the arcuate complex which consists of the popliteofibular ligament, the arcuate ligament, and the variably present fabellofibular ligament [1]. Proximal fibula fracture represents an injury to the posterior lateral corner (PLC) of the knee, a primary stabilizer of varus stress, external tibial rotation, and posterior tibial translation (3). The repairing of the proximal fibula injury can reconstruct the stability of the posterolateral structures of the knee.

About 1/3 of tibial plateau fractures are combined with proximal fibula fractures, and bone fragment morphology greatly affects the choice of surgical approach and fixation strategy of the tibial plateau fracture. More than 40 kinds of tibial plateau fractures have not been paid enough attention to the often-concomitant proximal fibula fractures. The widely used Schatzker classification 1974 and AO classification 2007 did not mention the fibular fracture [2, 3]. Luo's three-column concept and Schatzker 2018 supplementary classification marked the anterior point of the fibular head as a landmark point merely [4, 5]. A comprehensive and practical classification of proximal fibula fractures associated with tibial plateau fracture is strongly needed.

Focus on the local, Bozkurt et al. first described the classification of proximal fibula combined with TPF in 2005 (type 1, involved avulsion fractures; type 2, fibula head fractures and/or fracture associated with proximal tibiofibular joint; type 3, fibular neck or head and neck fractures; and type 4, fibula proximal diaphysis fractures) [6]. In 2018, we proposed a comprehensive “four-column and nine-segment” classification that described the injury types of the proximal tibia and fibula. We first named the bony and soft tissue structure of proximal fibula as “fibular column” and defined the six new-style injury types [7]. In the AO classification 2018 edition, the proximal fibula fracture was coded independently as 4F1A/4F1B (qualifications: extra-articular or intra-articular) and divided into the simple fracture and multifragmentary fracture [8]. In 2019, Zheng et al. divided this special fracture into five patterns according to fracture line and degree of comminution (avulsion fractures, fibular head cleavage fractures, fibular head depressed fractures, comminuted fractures, and fibular neck or shaft fractures). More precisely, Cohen et al. classified the avulsion of fibular head into three subtypes (fibular styloid, fibular head which involves the arcuate complex, and fibular head which involves the metaphyseal) in 2018 [9].

However, there is no literature summarizing the injury mechanism and treatment strategies for this special fracture according to certain classifications to date. The four-column nine-segment classification (Yao classification) seems to be more comprehensive and clear in the current classifications. The aim of this study was to explain the precise treatment strategy of concomitant fibular column injuries in TPFs depending on the four-column nine-segment classification.

## 2. Methods

After approval was obtained from the authors' institution's human subject review board, medical records, including digital radiologic data of all the patients treated for tibial plateau fracture from Aug 2007 to March 2020, were reviewed. On admission to the hospital, anteroposterior (AP) and lateral X-ray and 3D computerized tomography (CT) images of the patients were obtained. Four observers, including 2 orthopedic trauma surgeons and 2 radiologists, reviewed the X-ray and computed tomography (CT) and on clinical picture archiving and communication system workstations. The fracture type was determined through consensus by the 4 observers. None of the observers had a conflict of interest regarding the patients. The exclusion criteria were age under 18 years, previous deformity, pathological fracture, inadequate imaging documentation, metabolic bone disease, or a history of knee surgery.

**Figure 1:**
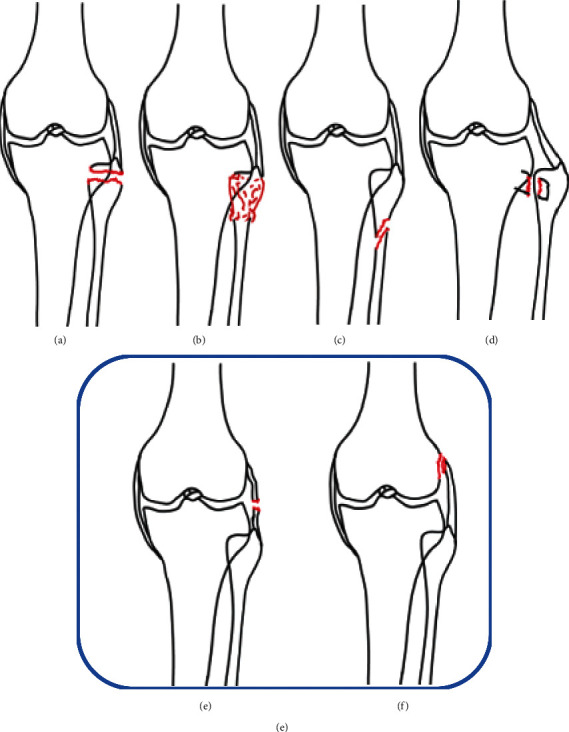
The six morphologies of fibular column injury in the four-column nine-segment classification: (a) type 1: avulsion fracture of fibular apex (“arcuate sign”); (b) type 2: fibular head fracture; (c) type 3: proximal diaphyseal fracture; (d) type 4: proximal tibiofibular joint dislocation; (e) expanding type 1 (EX1): LCL tear; (f) expanding type 2 (EX2): avulsion fracture of lateral femoral condyle.

All the tibial plateau fractures were classified according to the AO and “four-column and nine-segment” classification. The tibial plateau injury index (TPII) and treatment scheme (surgically treated or conservatively treated) were also recorded. The six injury types of the fibular column (segment i) were the following: type 1: avulsion fracture of the fibular apex (“arcuate sign”), type 2: fibular head fracture, type 3: proximal diaphyseal fracture, and type 4: proximal tibiofibular joint dislocation. The two expanding types were the following: EX1: LCL tear and EX2: avulsion fracture of the lateral femoral condyle (Figure 1).

### 2.1. Statistics

Statistical analysis was carried out using IBM SPSS Statistics 19 (SPSS Inc, Chicago, USA). Qualitative data are shown as *n* (percentage), and quantitative data are expressed as the mean ± SD. A two-sided *t*-test was used to determine the significance of differences between the gender and sides. *P* < 0.05 was considered statistically significant.

## 3. Results

### 3.1. Results

Totally, 1160 patients with 1170 knees suffering TPFs were retrospectively analyzed. Thereinto, 352 patients with 355 knees (355/1170, 30.3%) combined tibial plateau fractures with proximal fibular injury (types 1 to 4) were enrolled in this study. Of these 352 patients, 192 were men and 160 were women with 1 male patient and 2 female patients involved with bilateral tibial plateau fractures. The average age was 53.9 ± 13.0 years (male/female 52.6 ± 12.8/56.1 ± 12.8 years) (Table 1). The cause of injury included electric bicycle and motorcycle accident, car accident, fall from a height, and sports injury.

According to the AO/OTA classification, there were 31 patients with type A (8.7%), 127 patients with type B (35.7%), and 197 patients with type C (55.4%). According to the Yao classification, there were 85 patients with 2 columns (23.9%), 149 patients with 3 columns (41.8%), and 121 patients with 4 columns (33.8%). TPII is 7.9 ± 2.6 (Table 1). Intermedial column and lateral column injury was the most, and the medial column was the least (Table 2).

Of the four injury types to the proximal fibula, type 2 fibular head fracture (193/355, 54.4%) was the most common. Type 1 fibular apex (122/355, 34.4%) was the second, and the third was the 37 (10.4%) cases of the type 3 fracture; only 3 (0.8%) cases of type 4 were found. EX1 was omitted for invisibility in CT, EX2 has 21 cases, and the proportion was 1.8% (21/1170).

In type 1, 38 patients needed surgery and 84 patients were conservative. In type 2, 18 patients needed surgery and 175 patients were conservative. In type 3, 3 patients needed surgery and 34 patients were conservative. In type 4, 2 patients needed surgery and 1 patient was conservative. In type EX2, 6 patients needed surgery and 15 patients were conservative (Table 3).

## 4. Discussion

In this study, we analyzed the incidence and treatment strategies of the largest sample size of concomitant fibular column injuries in TPFs. The current study demonstrated that 30.3% (355/1170) of tibial plateau fractures were associated with proximal fibula fractures, which was in accordance with previous studies [10]. The prevalence of proximal fibular fractures associated with tibial plateau fracture was 22.2%-38.3% [6, 11, 12].

Among the four injury types, the type 2 fibular head compression fracture (193/355, 54.4%) was the most common, the type 1 avulsion fracture of the fibular head (122/355, 34.4%) was the second, and type 3 the proximal diaphyseal fracture was the least. Only three cases were found in the proximal tibiofibular joint dislocation, which was a severe traumatic marker for neurovascular injury and compartment syndrome [3].

In terms of injury type, in the Yao classification, type 1 means the avulsion of fibular apex or head (arcuate fracture or arcuate sign) just consistent with type 1 injury in Bozkurt classification and Zheng classification. In Yao classification, type 2 compressed fibular head fracture contained Bozkurt classification type 2 + 3 and Zheng classification type 2 + 3 + 4, regardless of the morphology of the fracture (cleavage, depressed, or comminuted). In the Yao classification, type 3 equaled to type 4 in Bozkurt classification and type 5 in Zheng classification.

In addition to the above three common injury types, Yao classification defined three neostyle injury types: type 4, EX1, and EX2. Uncommon type 4 (3/355, 0.85%) was a severe traumatic marker for neurovascular injury and compartment syndrome [6]. EX1 was imponderable in this study, and 21 cases (21/1170, 1.8%) of EX2 of the fibular column were found. It is the first time that LCL tear (EX1) and avulsion of the lateral femoral condyle (EX2) are considered fibular column injury type in any classification for the proximal calf.

**Figure 2:**
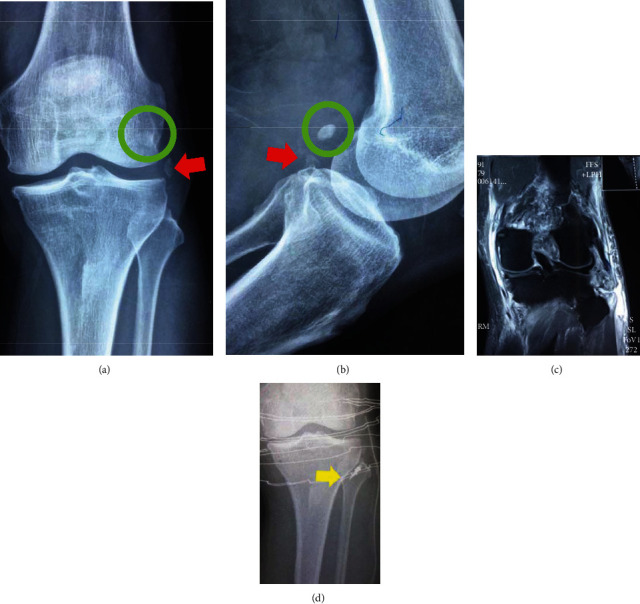
A type 1 fibular column injury case: (a, b) the preoperative X-ray; (c) the MRI shows avulsion of lateral collateral ligament; (d) the avulsion fracture of fibular apex was fixed by suture anchor. Green cycle: sesamoid; red arrow: avulsion fracture of fibular apex; yellow arrow: metal anchor.

Based on the Yao classification, type 1 avulsion of fibular apex (head) fracture is more common in tension injury and is usually caused by varus/hyperextension force. Fixation of avulsion of the fibula head is important for the restoration of stability of the posterolateral corner. The smaller and undisplaced avulsions are more likely to be treated conservatively, while the bigger and displaced fragment required fixation/repair or reconstruction. In the cohort, 38 of 122 cases underwent surgical treatment. The recommended fixation scheme included lag screw ± washer, suture tunnel, and suture anchor technique [13–15].  Figure 2 shows a type 1 case fixed by the suture anchor technique.

**Figure 3:**
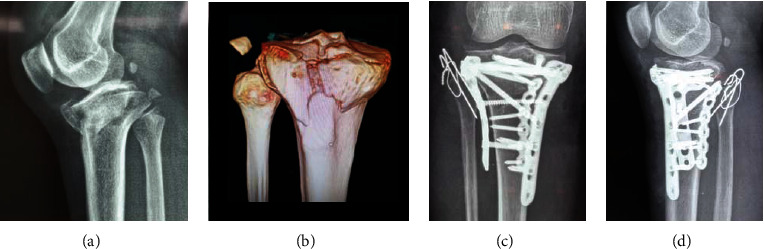
A type 1 case fixed with Kirschner wire by tension band technique: (a) preoperative lateral X-ray; (b) preoperative CT; (c, d) preoperative X-ray.

Kirschner wire and tension band technique or locking plates only are not recommended for spontaneous loosening and lack of effective compression (Figure 3).

**Figure 4:**
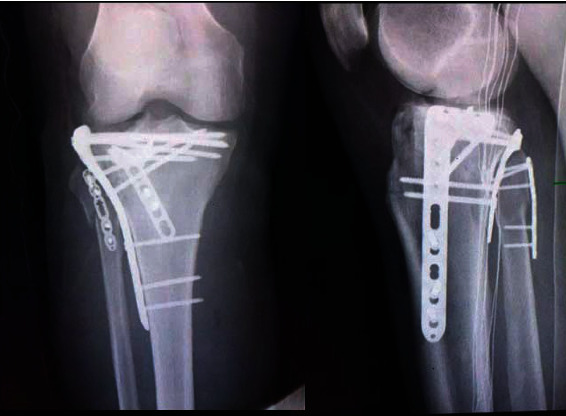
A type 2 case fixed by a mini locking plate: (a, b) preoperative X-ray.

The type 2 fibular head compression fracture is highly related to the mechanism of valgus or compression injury of the knee. The impacted fracture site is relatively stable, and most of this injury just required conservative treatment. If it is obviously unstable, it can also be fixed with a screw and plate (Figure 4).

**Figure 5:**
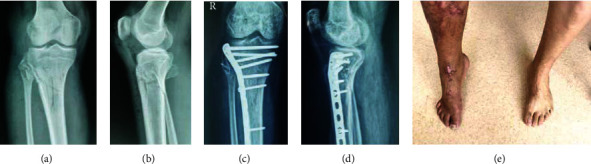
A tibial plateau fracture case with type 2 fibular column injury fixed with a steel wire. The patient showed iatrogenic peroneal nerve injury with foot drop and paresthesia postoperatively: (a, b) preoperative X-ray; (c, d) postoperative X-ray; (e) the symptom of right foot drop.

There is the possibility of iatrogenic injury of the common peroneal nerve in the operation of the peroneal head and neck. Less than 10% (15/193) of type 2 cases received surgical treatment.  Figure 5 shows 1 TPF patient with fibular type 2 that underwent a fix using steel wire. Unfortunately, the patient developed iatrogenic foot drop and sensory disturbance of common peroneal injury after the operation.

**Figure 6:**
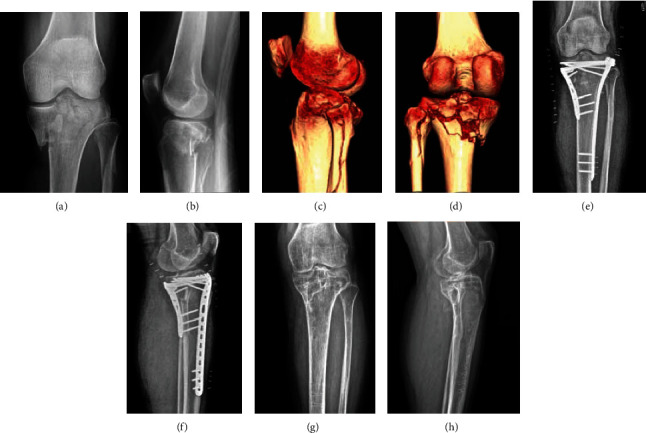
A tibial plateau fracture case with type 3 fibular column injury: (a, b) preoperative X-ray; (c, d) preoperative CT; (e, f) postoperative X-ray; (g, h) X-rays after removal of implant.

The incidence of type 3 injury is low (37/355) which is difficult to expound by a certain injury mechanism. The elastic intramedullary pin scheme which does not need to expose the nerve could be an option worth considering.  Figure 6 shows a TPF case with type 3 fibular column injury.

**Figure 7:**
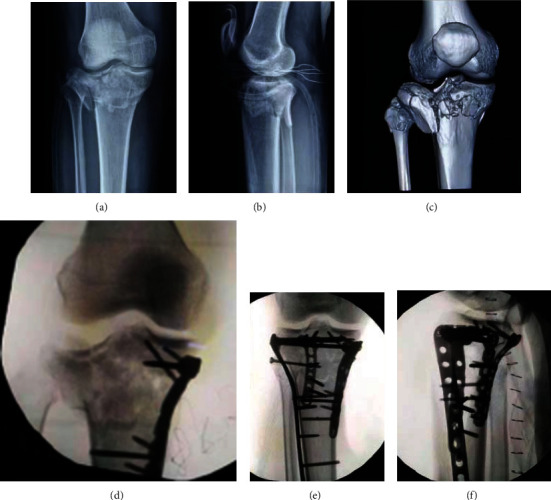
A tibial plateau fracture case combined with a superior tibiofibular dislocation (type 4 injury in four-column nine-segment classification) was fixed by plates and a positioning screw: (a) preoperative A-P view X-ray; (b) preoperative lateral-view X-ray; (c) preoperative three-dimensional CT imaging; (d) intraoperative A-P view X-ray showed the obvious dislocation of the superior tibiofibular joint; (e) intraoperative A-P view X-ray after the placement of the positioning screw; (f) intraoperative lateral-view X-ray.

Only three (3/355, 0.85%) cases with type 4 proximal tibiofibular joint dislocation were found in this cohort. The proximal tibiofibular joint dislocation was first described by Ogden in 1974 [16]. It is difficult to be identified on X-ray, and 3D-CT can effectively identify the separated proximal fibula. A hook test could be used to judge the stability during operation similar to the inferior tibiofibular syndesmosis. For fresh dislocation, screw, suture button, and tight rope could be used to restore the proximal tibiofibular joint [17–19]. Temporary screw fixation could be utilized but requires removal of the screws at 12 to 16 weeks postoperatively.  Figure 7 shows a TPF case with type 4 fibular column injury; a positioning screw was placed to maintain the stability of the proximal tibiofibular joint. Delayed dislocation of the superior tibiofibular syndesmosis may require ligament reconstruction [20, 21].

**Figure 8:**
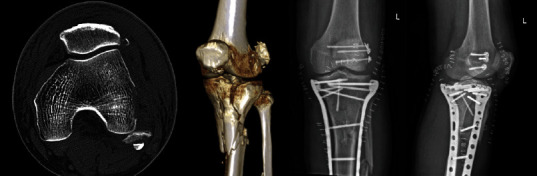
A case of extending type 2 (EX2) avulsion of lateral femoral condyle fixed by lag screws: (a) preoperative axial view CT imaging; (b) preoperative three-dimensional CT imaging; (c, d) postoperative A-P view X-ray.

Both extending types (EX1 and EX2) covered the injury information about LCL (PLC) and originated from varus/extension/hypertension injury the same as type 1 avulsion. For EX1, early repair or primary reconstruction brings about a better prognosis [22]. Missed EX1 injury could lead to sustained posterolateral instability, which required arthroscopic or open reconstruction [23, 24]. All avulsion of lateral femoral condyle could be recorded as EX2 of the fibular column which is usually considered LCL avulsion injury. Small and undisplaced bony pieces are usually conservative while large pieces of bone need to be fixed surgically (Figure 8).

**Figure 9:**
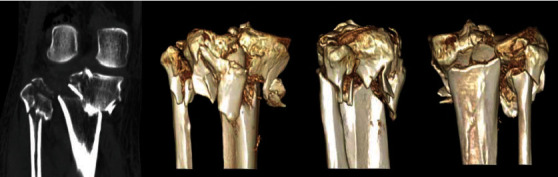
A tibial plateau fracture case with type 1 + type 2 fibular column injury.

Obviously, the setting of six categories in four-column nine-segment classification (Yao classification) can effectively remind orthopedic surgeons to avoid missing various injury patterns from the lateral condyle of the femur to the fibular shaft. It should be noted that the injury type is only a morphological description of the distribution of fracture lines. A certain kind may originate from violence at different levels and in different directions. For example, an undisplaced fibula tip crack fracture can be caused by mild tension or compression violence. Moreover, six kinds of injury types do not exist alone and occasionally coexist.  Figure 9 illustrates a TPF case with type 1 + type 2 fibular column injury.

There were several limitations in this study. First, although our sample size is by far the largest, increased samples and multicenter study will lead to a more accurate incidence. Second, the incidence of EX1 has not been revealed for lacking MR or ultrasound data, and MR will provide injury information of the total knee joint more than the posterolateral corner. Third, the relationship between the injury type of the fibular column and the functional recovery of the knee needs further follow-up and summarization.

## 5. Conclusions

Six injury types in the four-column nine-segment classification covered all patterns of bony and soft tissue injuries of the fibular column and concisely explained the injury mechanism. The classification is helpful for the precise judgement and decision-making of the concomitant fibular column injuries in tibial plateau fractures.

## Figures and Tables

**Table 1:** Demographic characteristics of patients and fractures.

	Characteristics	
Patients (*n* = 352)	Sex ratio (male/female)	192/160 (1 : 0.83)
	Age (±SD, years)	53.9 ± 13.0
	Age (±SD, years; male/female)	52.6 ± 12.8/56.1 ± 12.8
Knees (*n* = 355)	Left only	207 (58.8%)
	Right only	142 (40.3%)
	Bilateral	3 (0.9%)
	Left/right	210/145 (1 : 0.69)
AO/OTA classification	41.A	31 (8.7%)
	41.B	127 (35.7%)
	41.C	197 (55.4%)
Columns involved (*n* = 355)	1 column	—
	2 columns	85 (23.9%)
	3 columns	149 (41.8%)
	4 columns	121 (33.8%)
TPII (*n* = 355)	Total	7.9 ± 2.6
	Columns	3.1 ± 0.8
	Segments	4.8 ± 2.0
	Male/female	7.9 ± 2.7/8.0 ± 2.6
	Left/right	7.6 ± 2.5/8.0 ± 2.7
	Mild comminuted (2-5)	84 (23.6%)
	Moderate comminuted (6-9)	174 (48.8%)
	Severe comminuted (10-13)	97 (27.1%)

**Table 2:** Morphology of fractures according to the four-column nine-segment classification.

Column		Segment	
Medial	146	a	109
		b	103
Intermedial	291	c	77
		d	196
		e	177
		f	182
Lateral	313	g	271
		h	246
Fibular	355	i	355

**Table 3:** The morphology of fibular column injury in the four-column nine-segment classification and related treatment.

	Injury type		Surgically treated	Conservatively treated
Fibular column	Type 1	122	38	84
	Type 2	193	15	178
	Type 3	37	3	34
	Type 4	3	2	1
	Total	355	58	297
	EX1	—		
	EX2	21/1170	6	15

EX1: extending type 1; EX2: extending type 2; “—”: data not available.

## Data Availability

The main object of this study is X-ray and CT image data, and we have provided a typical illustrative diagram. The extra data used to support the findings of the current study are included within the article.
